# Comparative Study of Prions in Iatrogenic and Sporadic Creutzfeldt-Jakob Disease

**DOI:** 10.4172/2155-9899.1000240

**Published:** 2014-07-29

**Authors:** Xiangzhu Xiao, Jue Yuan, Liuting Qing, Ignazio Cali, Jacqueline Mikol, Marie-Bernadette Delisle, Emmanuelle Uro-Coste, Liang Zeng, Mai Abouelsaad, Dimitris Gazgalis, Manuel Camacho Martinez, Gong-Xian Wang, Paul Brown, James W. Ironside, Pierluigi Gambetti, Qingzhong Kong, Wen-Quan Zou

**Affiliations:** 1 Department of Pathology and National Prion Disease, Pathology Surveillance Center, Case Western Reserve University, 2085 Adelbert Road, Cleveland, Ohio 44106, USA; 2 Department of Pathology, Hôpital Lariboisière, 2 rue Ambroise Paré, Paris, France; 3 Department of Pathology, Rangueil University Hospital, avenue Jean Poulhes, TSA 50032, 31059 Toulouse Cedex 9, France; 4 INSERM U858, I2MR, Team 15, BP 84225, 31432 Toulouse Cedex 4, France; 5 Laboratoire Français des Biotechnologies (LFB), Les Ulis, France; 6 National Creutzfeldt-Jakob Disease Surveillance Unit, Western General Hospital Edinburgh, EH4 2XU, United Kingdom; 7 Department of Neurology, Case Western Reserve University, 2085 Adelbert Road, Cleveland, Ohio 44106, USA; 8 National Center for Regenerative Medicine, Case Western Reserve University, 2085 Adelbert Road, Cleveland, Ohio 44106, USA; 9 The First Affiliated Hospital, Nanchang University, Nanchang, Jiangxi Province, The People’s Republic of China; 10 Department of Clinical and Experimental Medicine, Second University of Naples, Naples, Italy

**Keywords:** Human prion diseases, Iatrogenic Creutzfeldt-Jakob disease, Sporadic CJD

## Abstract

Differentiating iatrogenic Creutzfeldt-Jakob disease (iCJD) from sporadic CJD (sCJD) would be useful for the identification and prevention of human-to-human prion transmission. Currently, the diagnosis of iCJD depends on identification of a recognized source of contamination to which patients have been exposed, in addition to fulfilling basic requirements for the establishment of diagnosis of CJD. Attempts to identify differences in clinical manifestations, neuropathological changes and pathological prion protein (PrP^Sc^) between iCJD and sCJD have been unsuccessful. In the present study, using a variety of more sophisticated methods including sucrose step gradient sedimentation, conformational stability immunoassay, protein misfolding cyclic amplification (PMCA), fragment-mapping, and transmission study, we show no significant differences in gel profiles, oligomeric state, conformational stability and infectivity of PrP^Sc^ between iCJD and sCJD. However, using PMCA, we find that convertibility and amplification efficiency of PrP^Sc^ is greater in iCJD than in sCJD in a polymorphism-dependent manner. Moreover, two protease-resistant PrP C-terminal fragments (termed PrP-CTF12/13) were detected in all 9 cases of sCJD but not in 6 of 8 cases of iCJD tested in this study. The use of fragment mapping- and PMCA-based assays thus provides a means to distinguish most cases of iCJD from sCJD.

## Introduction

Human prion diseases are highly heterogeneous and can be classified as sporadic, hereditary or acquired. Although all of them are associated with a pathological prion protein (PrP^Sc^) that assimilates its cellular form (PrP^C^) by a conformational transition, the PrP^Sc^ origin in acquired prion disease is apparently different from that of sporadic and hereditary forms in which PrP^Sc^ is formed spontaneously. Acquired prion diseases including iatrogenic Creutzfeldt-Jakob disease (iCJD), variant CJD (vCJD) and kuru are caused by exposure to the infectious prions. Variant CJD results from the consumption of beef products contaminated by bovine spongiform encephalopathy (BSE) and is characterized by the deposition in the affected brain of an exclusive single type of BSE-derived PrP^Sc^. Iatrogenic infections have been associated with corneal transplant (n=2), stereotactic EEG (n=2), neurosurgery (n=4), dura mater graft (n=228), growth hormone (n=226), gonadotropin (n=4) and blood transfusion (n=4) [1,2].

The clinical presentations of iCJD may differ from those of sCJD due to mode and route of infection. Patients with infection that was introduced directly into the substance of the brain (either via neurosurgical or EEG electrodes) showed shorter incubation periods and mental deterioration, while patients with infection that was introduced via brain surface (i.e. dura matter graft) or a peripheral route (i.e. human pituitary hormones) revealed much longer incubation times and cerebellar signs [3]. However, it is impossible to differentiate iCJD from sCJD based on the clinical manifestations. In addition, as in sCJD, homozygotes at codon 129 of PRNP seem to be predisposed to the disease [3,4]. 102 of 128 iCJD cases were homozygous at codon 129 (~80%): 73/128 for methionine/methionine (MM) homozygosity (~60%) and 29/128 for valine/valine (VV) homozygosity (~20%), while only 26/128 were MV heterozygous (~20%) [3,4].

Regarding molecular typing of PrP^Sc^ from iCJD, there have been discrepancies between the Parchi/Gambetti and Collinge classifications, a situation that also occurs in sCJD. Of seven cases reported by Collinge, three VV associated with growth hormone, one VV with gonadotropin; and an MV with growth hormone were all categorized as type 3, the remaining 2 cases were both MM (one growth hormone type 1 and one dura graft type 2) [5]. In contrast, of seven iCJD cases examined by Parchi and co-workers, four MM cases associated with dura, electrode, corneal transplant, or growth hormone, respectively, were type 1; and three homozygous VV cases (contamination source unknown) were type 2 [6–8]. No distinguishable differences in gel mobility and glycoform ratios of PrP^Sc^ between iCJD and sCJD were observed by either group [5,6]. It is thus evident that traditional molecular typing of PrP^Sc^ is incapable of distinguishing iCJD from sCJD.

The distinction remains important because cases continue to occur in which the clinical history and illness of CJD are compatible with either a sporadic or environmental origin of disease, and if environmental may implicate a wider contamination of already known sources of infection, or even the more worrisome identification of previously unrecognized sources. For example, the recent discovery of several blood transfusion-associated vCJD infections has raised new concerns about the possibility of further secondary transmissions from operative procedures as well as blood and tissue donations [9–12], and the occasional case of CJD associated with potentially contaminated neurosurgical procedures can be extremely difficult to evaluate and identify [2,3,13].

The goal of our study was to extend the search for physicochemical and biological features that could provide a more secure distinction between iCJD from sCJD. We investigated the oligomeric state, convertibility by in vitro cell-free amplification, enzymatic fragmentation and animal infectivity of PrP^Sc^. We found no differences in gel profiles, oligomeric states and transmissibility of PrP^Sc^ between iCJD and sCJD; however, the efficiency of protein misfolding cyclic amplification (PMCA) [14,15] is higher in iCJD than in sCJD, and that most iCJD (and all vCJD) did not show the PK-resistant C-terminal fragments detectable in sCJD.

## Patients and Methods

### Patient brain collections

Brain tissues obtained at autopsy from iatrogenic, sporadic or variant CJD cases were collected in the United States (National Prion Disease Pathology Surveillance Center, Cleveland, OH), France (Departments of Pathology, Paris and Toulouse), or United Kingdom (National CJD Research & Surveillance Unit University of Edinburgh Western General Hospital Edinburgh), respectively, and were frozen at −80°C or fixed with 10% formalin. Frozen brain tissues from eight iCJD, nine sCJD and three vCJD were used in this study. Seven out of eight iCJD cases were associated with human growth hormone while one was associated with a dura matter graft. Written consent to use autopsy material for research purposes was obtained from patients or legal guardians for all samples. Clinical data and relevant hospital records were coded and handled according to the protocols approved by the Ethical Committee and Institutional Review Board of Case Western Reserve University to protect patients’ identities.

### Molecular genetics

The genomic DNA was extracted from frozen brain tissues. The ORF of the human prion protein gene PRNP was amplified by the polymerase chain reaction (PCR). The PCR products were subjected to automated sequencing. They were cloned then sequenced to confirm the mutation and polymorphisms as previously described [16].

### Neuropathological studies

The following procedures were performed as described [6]. For histological preparations, brain sections were embedded in paraffin and stained with hematoxylin-eosin. For immunohistochemistry, sections were deparaffinized, rehydrated, and immersed in 98% formic acid for 1 h at room temperature. Endogenous peroxidase was blocked by immersion in 8% hydrogen peroxide in methanol for 10 min. Sections were completely immersed in 1.5 mM hydrochloric acid and microwaved for 10 min. After rinsing, they were incubated with the mouse monoclonal antibody 3F4 at 1:600, washed and incubated with bridge antibody (goat anti-mouse, Cappel, 1:50) followed by incubation with mouse PAP complex (Sternberger, Meyer Immunocytochemicals, 1:250). Diaminobenzidine tetrahydrochloride was used to visualize the immunoreactivity [6].

### Preparation of brain homogenate, proteinase K-digestion and deglycosylation of PrP

The 10% (w/v) brain homogenates were prepared in 9 volumes of lysis buffer (10 mM Tris, 150 mM NaCl, 0.5% Nonidet P-40, 0.5% deoxycholate, 5 mM EDTA, pH 7.4) with pestle on ice. For proteinase K (PK)-digestion of PrP, the samples were incubated with PK at 50 μg/ml, or 100 μg/ml as designated at 37°C for 1 h. For deglycosylation of the protein, the brain homogenates were denatured and incubated in the presence of recombinant peptide N-glycosidase F (PNGase F) according to the manufacturer’s protocol (Roche Molecular Biochemicals).

### Sucrose step gradient sedimentation

Brain homogenates (20%, w/v) in 1X Dulbecco’s PBS pH 7.4 were mixed with an equal volume of 2X lysis buffer, then centrifuged for 10 min at 1,000 × g at 4°C. Supernatants were collected and sarkosyl was added to 1% final concentration. Each sample was loaded atop of 10–60% step sucrose gradients and centrifuged at 200,000 × g in a SW55 rotor for 1 h at 4°C as described with minor modification [17,18]. After centrifugation, the content of the centrifuge tubes was sequentially removed from the top to collect 11 fractions which were subjected to immunoblotting as described below.

### Gel filtration

Gel filtration, also called size exclusion chromatography, was performed as previously described [17,18]. In brief, Superdex 200 HR beads (GE, Healthcare Biosciences, Pittsburgh, PA, USA) in a 1 × 30 cm column were used to determine the oligomeric state of PrP^Sc^. Chromatography was performed in an FPLC (Fast protein liquid chromatography) system (GE, Healthcare Biosciences, Pittsburgh, PA, USA) at a flow rate of 300 μl/min, Fractions of 300 μl were collected. The molecular weight (MW) of the PrP complexes recovered in different FPLC fractions was determined using a calibration curve generated with the gel filtration of molecular mass markers (Sigma, St. Louis, MI) including Dextran blue (2,000 kDa), thyroglobulin (669 kDa), apoferritin (443 kDa), β-amylase (200 kDa), alcohol dehydrogenase (150 kDa), albumin (66 kDa), and carbonic anhydrase (29 kDa). These standards were loaded independently at the concentrations recommended by Sigma in 200 μl sample volumes. To determine the distribution of PrP in FPLC fractions, 150 μl from each fraction was concentrated by incubating with 4-fold pre-chilled methanol at −20°C for 2 h followed by centrifugation at 16,000 × g for 30 min at 4°C. After discarding the supernatant, the pellet from each fraction was re-suspended in 20 μl of sample buffer and was analyzed by Western blotting.

### Conformational stability immunoassay of PrP^Sc^

Aliquots of brain homogenates were incubated with various concentrations of guanidine hydrochloride (GdnHCl) from 0 to 3.0 M [19,20]. Following incubation for 1 h at room temperature, all samples were precipitated in pre-chilled methanol at −20°C for 2 h and centrifuged at 16,000 g for 30 min at 4°C. Pellets were resuspended in 1 x lysis buffer and treated with 100 μg/ml PK for 1 h at 37°C. The reaction was stopped by adding phenylmethylsulfonyl fluoride (3 mM) and gel loading buffer and boiled for 10 min. The samples were used for Western blot analysis as described below.

### Protein misfolding cyclic amplification (PMCA)

PMCA was performed as described with slight modifications [15,21]. In brief, 99 μl of brain homogenate from uninfected transgenic (Tg) mouse brains expressing human PrP-129M (Tg40h) as a substrate was incubated with 1 μl of brain homogenate from iCJD or sCJD patients. A portion was removed and frozen at −80°C as the non-PMCA-treated sample controls. The remaining sample (~80 μl) in a 0.2 μl PCR tube was placed on a microplate horn filled with water and subjected to PMCA, consisting of cycles of 30 min incubation at 37°C followed by a 40-second pulse of sonication at 60% potency for 18 h in a sonicator (QSONICA 700, Newtown, CT). To detect the amplified PrP^Sc^, 20 μl of PMCA treated or untreated sample was incubated with 100 μg/ml PK for 70 min at 45°C. The reaction was terminated by adding PMSF to a final concentration of 5 mM and an equal amount of SDS sample buffer. Samples were then heated at 100°C for 10 min and a 10 μl sample was subjected to SDS-PAGE and Western blotting with 3F4. Serial PMCA was conducted as previously described [15], with a minor modification. In brief, 10 μl of PMCA-treated sample taking from a previous round of PMCA to service as seeds was transferred to 90 μl fresh Tg40 mouse brain homogenate servicing as substrates. And after taking 20 μl for non-PMCA control, the remaining sample was subjected to PMCA as described above. This process was repeated up to 10 rounds.

### Transmission study

Infectivity of PrP^Sc^ was evaluated by inoculation of Tg mice expressing human PrP^C^-129M (Tg40h), at 2x the wild-type level in the mouse PrP-ablated background, with PrP^Sc^-containing brain homogenates from iCJD or sCJD as previously described [22]. Briefly, after anesthetization with isoflurane, 30 μl of 1% brain homogenate (in PBS) from sCJDMM1 or iCJDMM with type 1-like PrP^Sc^ was injected into each mouse brain intracerebrally (i.c.) with a 26-gauge needle inserted to a depth of 4 mm at the left parietal region of the cranium. The mice were monitored for prion-related signs every other day and sacrificed 2 or 3 days after the appearance of severe signs or at death. The brains were collected and sliced sagittally, with half frozen for immunochemical studies and the other half fixed in 10% formalin for histological and immunohistochemical staining analysis. Total PrP as well as PK-resistant PrP^Sc^ was determined by immunoblotting in sodium dodecyl sulfate (SDS)-polyacrylamide gels as described below. This study was conducted with approvals from the Institutional Review Board and the Institutional Animal Care and Use Committee.

### Sodium dodecyl sulfate-polyacrylamide gel electrophoresis (SDS-PAGE) and immunoblotting

Samples were mixed with an equal volume of 2 X SDS sample buffer and heated at 100°C for 10 min. Proteins were separated using 15% Tris-HCl pre-cast gels (Bio-Rad). After electrophoresis at 70 V for 2 h, proteins were electrotransferred onto the polyvinylidene difluoride (PVDF) membranes from the gel. The membranes were blocked with 5% non-fat milk in TBS-T buffer overnight at 4°C or 1 h at 37°C prior to incubation with different antibodies. Membrane-bound proteins were probed with anti-PrP antibodies 3F4 at 1:40,000 or anti-C at 1:6,000. The blot was then incubated with HRP-conjugated sheep anti-mouse antibody or donkey anti-rabbit antibody at 1:3,000. The PrP bands were visualized by enhanced chemiluminescence according to the manufacturer’s protocol (ECL Plus, Amersham Pharmacia Biotech, Inc., Piscataway, NJ).

### Statistical analysis

Statistical significance of differences in PrP intensity was evaluated using Student’s t-test. A difference was considered statistically significant if the p value was <0.05.

## Results

### Gel mobility and glycoform ratio of PK-resistant PrP^Sc^

**Figure 1:**
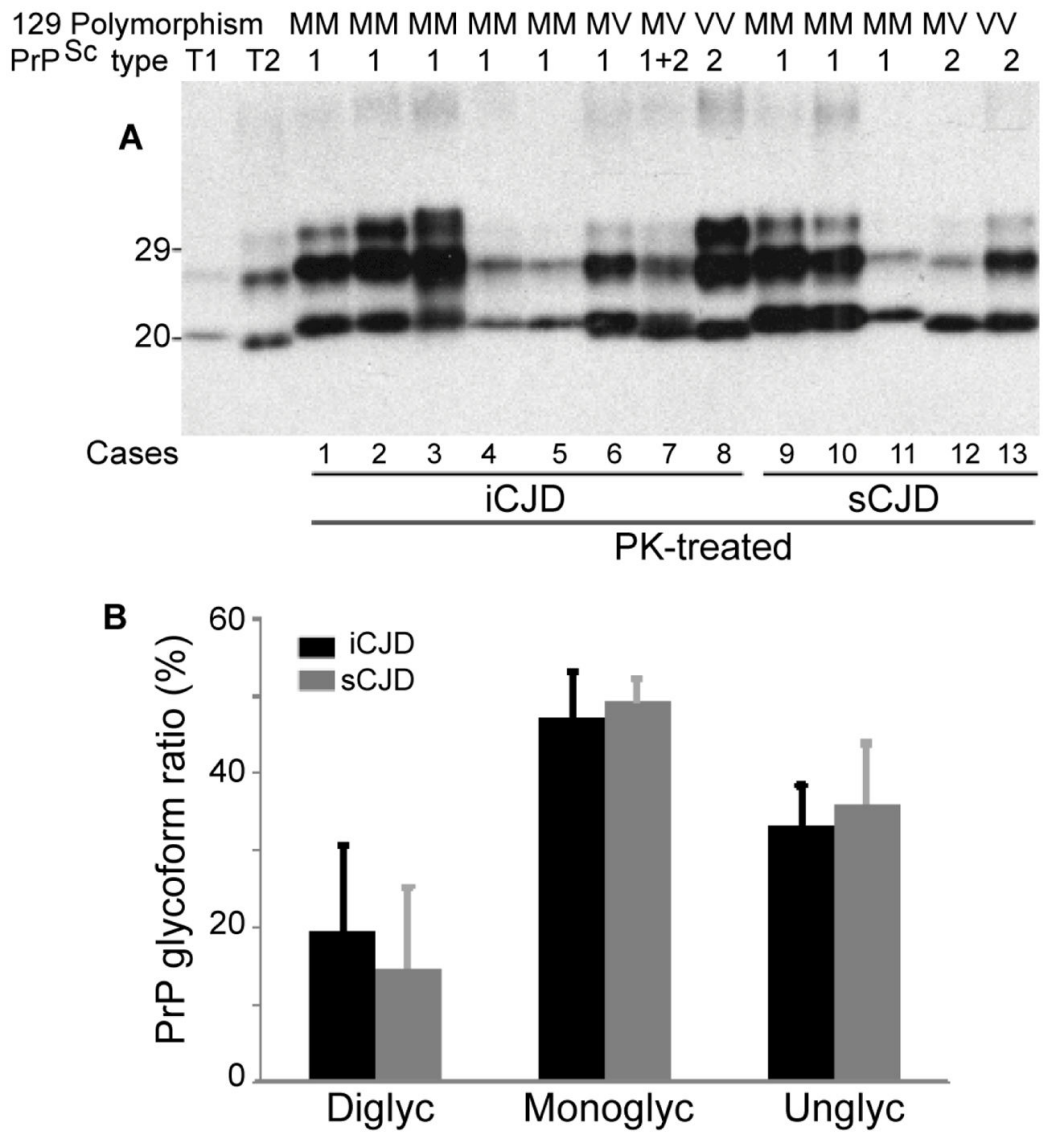
Comparison of gel mobility and glycoform ratio of PK-resistant PrP^Sc^ from iCJD and sCJD **(A)** Brain homogenates from eight iCJD cases and five sCJD cases were treated with PK (50 μg/ml) prior to Western blotting with 3F4. PRNP analysis shows that cases 1 through 5 are of homozygous MM, cases 6 and 7 heterozygous MV and case 8 homozygous VV polymorphism at residue 129. Cases 1 through 6 share with sCJDMM1 and case 7 with sCJDMV2 the gel mobility and glycoform ratio. The gel mobility and glycoform ratio of PK-resistant PrP^Sc^ from iCJD matched with those of sCJD. **(B)** Quantitative analysis of glycoform ratios of PrP^Sc^ from iCJDMM (n = 5) and sCJDMM (n = 3). There were no significant differences in glycoform ratios of PrP^Sc^ between iCJD and sCJD.

Using conventional Western blotting, we first investigated the gel mobility and glycoform ratio of PK-resistant PrP^Sc^ in the brain homogenates of iCJD and sCJD (Figure 1A). Brain homogenates from eight iCJD and five sCJD cases were treated with PK at 50 μg/ml prior to Western blotting analysis probing with 3F4. Human prion protein gene (PRNP) was analyzed to determine the MV polymorphism at codon 129 and to exclude PrP mutations. Of eight iCJD cases, five were MM homozygous, two MV heterozygous, and one VV homozygous. Five MM and one MV exhibited type 1-like PrP^Sc^ (iCJDMM1 or MV1) as well as one MV and one VV were type 2-like PrP^Sc^ (iCJDMV2 or VV2). No significant differences in gel mobility of PK-resistant PrP^Sc^ were observed between the two diseases (Figure 1A). Glycoform ratio of PK-resistant PrP^Sc^ was quantified and no significant differences were found [upper/middle/lower bands: 19.6 ± 11.0/47.2 ± 5.9/33.2 ± 5.2 (iCJD) vs. 14.8 ± 10.4/49.2 ± 3.0/36.0 ± 7.8 (sCJD)] (Figure 1B).

### PrP oligomeric state determined by sucrose step gradient sedimentation

**Figure 2:**
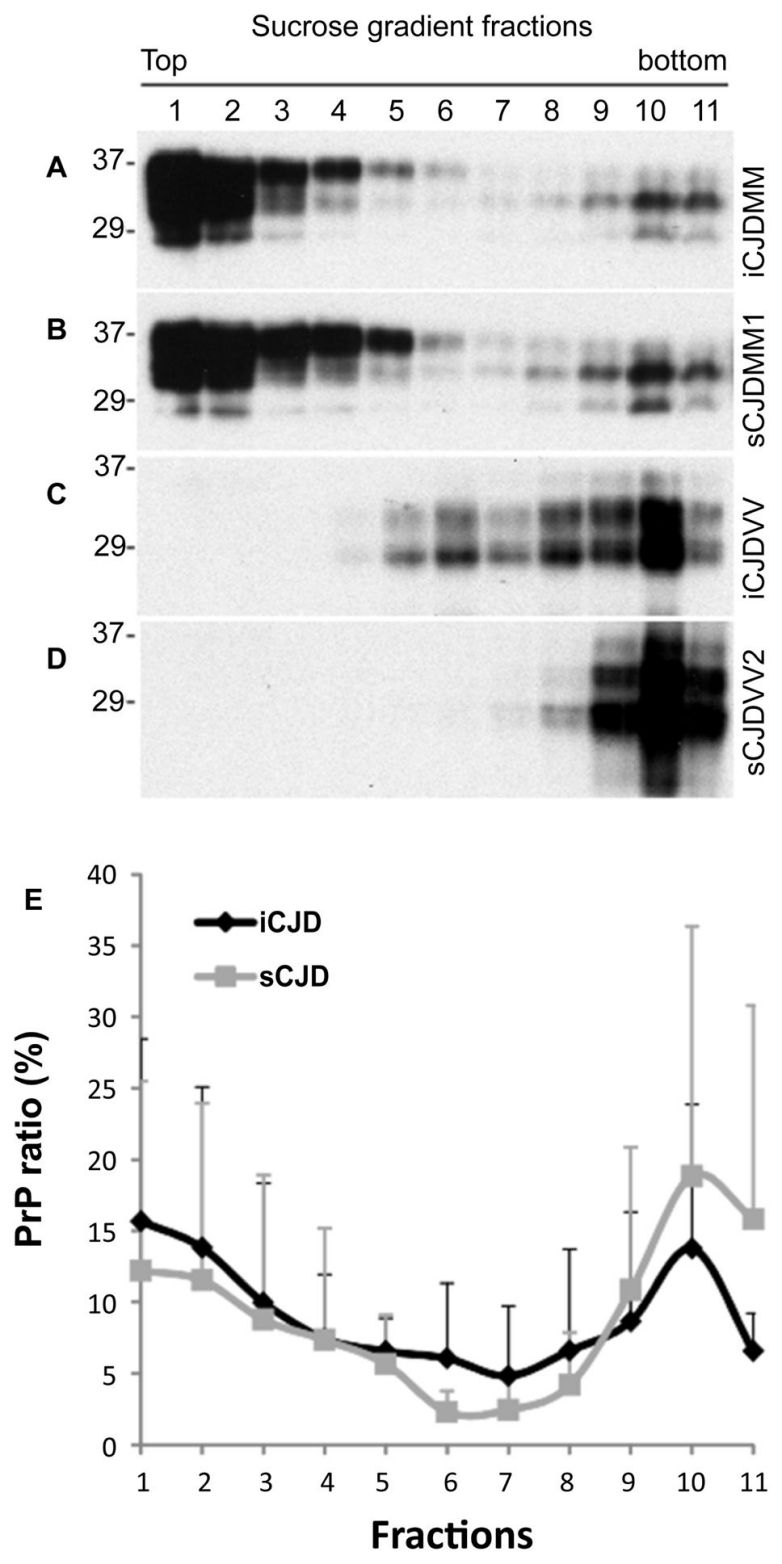
Analysis of PrP oligomeric state by sucrose step gradient sedimentation (A through D) Western blot analysis of PrP in different fractions of sucrose step gradient sedimentation. After ultracentrifugation of PrP in sucrose step gradients, 11 fractions were collected from the top of the gradients. PrP in individual fractions was detected by Western blotting with 3F4, which was subjected to densitometric analysis. **(A)** iCJDMM1; **(B)** sCJDMM1; **(C)** iCJDVV2; **(D)** sCJDVV2. PrP recovered in the top fractions 1–3 mainly represents monomers or small oligomers while PrP recovered in the bottom fractions 9–11 represents larger aggregates. **(E)** Ratio of PrP^Sc^ in each fraction to total PrP in all 11 fractions quantitated by densitometric analysis. No significant differences in PrP distribution profile were detected between iCJD (n=5) and sCJD (n=5).

After ultracentrifugation on sucrose gradients, the PrP molecules can be separated based on their size, shape, and density and this approach has been used to determine PrP oligomeric state [17,18]. PrP in eleven individual fractions was determined by Western blotting with 3F4 and quantified using a densitometric analysis. Like PrP from sCJDMM1 cases, PrP from iCJDMM1 cases was mainly recovered in the top factions 1–3 that represents monomers or small oligomers while a smaller amount of PrP was precipitated in the bottom fractions 9–11 that represents larger aggregates (Figures 2A and 2B). Likewise, like PrP from sCJDVV2 case, PrP from iCJDVV2 case was mainly recovered in the bottom fractions 9–11 (Figures 2C and 2D). Although more PrP was observed in fractions 5–8 in iCJDVV2 than sCJDVV2, no significant differences in the PrP distribution were found (n=5 for iCJD and sCJD group each) (Figure 2E), suggesting a similar PrP^Sc^ oligomeric state between the two conditions.

### PrP oligomeric state determined by gel filtration

**Figure 3:**
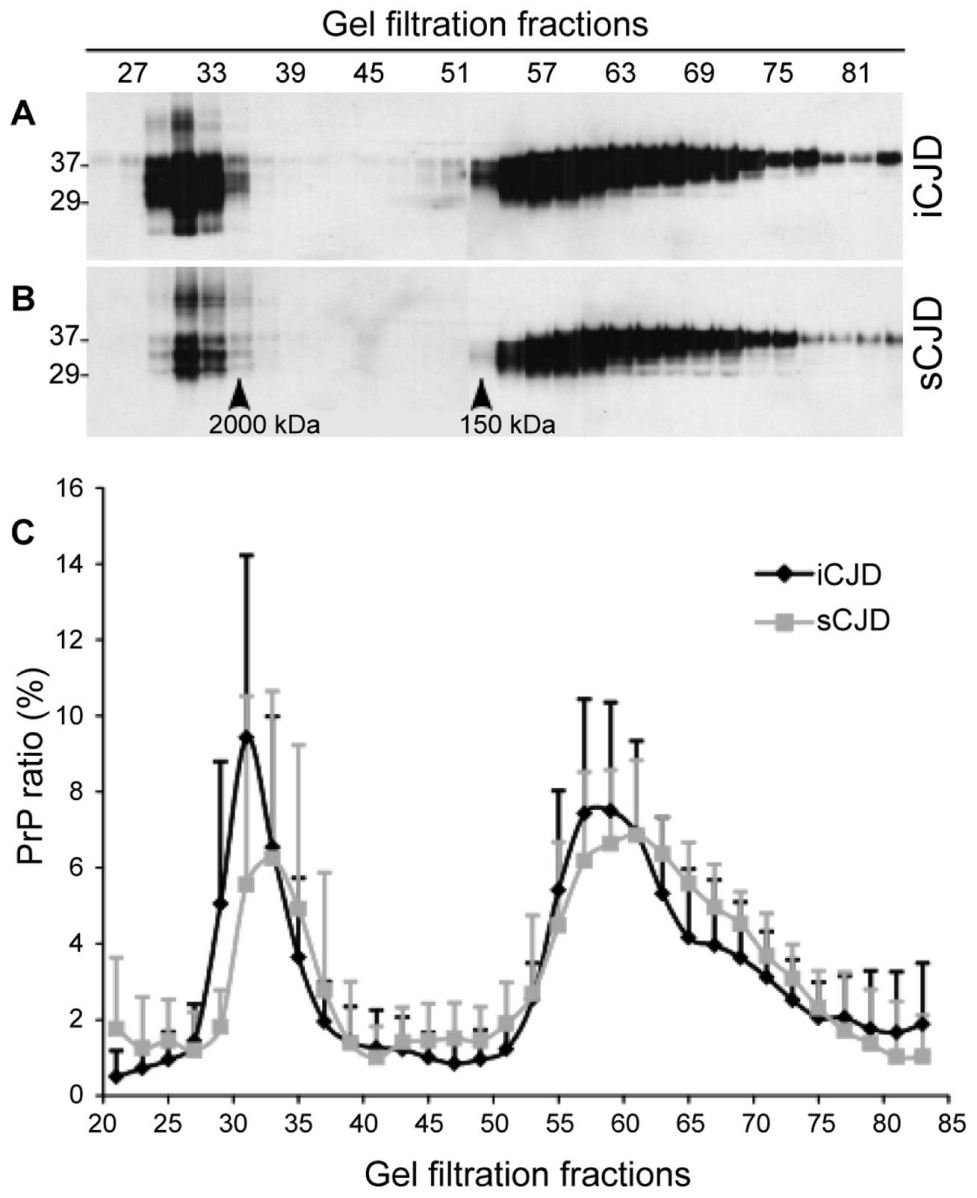
Analysis of PrP oligomeric state by gel filtration. Brain homogenates from three iCJD and three sCJD cases were subjected to gel filtration. **(A)** and **(B)** PrP in individual fractions was detected by Western blotting with 3F4. **(A)** iCJD; **(B)** sCJD. The amounts of total PrP in gel filtration fractions from 25 to 83 were determined by densitometric analysis. There were mainly two peaks of PrP distribution in these fractions: PrP in fractions between 29 and 35 represents large aggregates with molecular weight greater than 2,000 kDa while PrP in fractions between 51 and 81 represents small oligomers or monomers with molecular weight smaller than 150 kDa. **(C)** Ratio of PrP in each fraction to total PrP in all fractions quantitated by densitometric analysis. No significant differences in PrP distribution profile were determined between iCJD (n = 5) and sCJD (n = 7).

Gel filtration has been used to separate protein molecules based on their sizes and to determine the molecular weights of PrP aggregates [17,18]. We next compared the distribution of PrP in the fractions of gel filtration. Five iCJD and seven sCJD cases were examined and the amount of PrP in each gel filtration fraction was determined by Western blotting and quantified by the densitometric analysis. The percentage of PrP in each fraction from the two types of CJD was compared. Two major populations of PrP detected in both iCJD and sCJD brain samples exhibited molecular weight greater than 2,000 kDa and smaller than 150 kDa, respectively (Figures 3A and 3B). Although the amount of PrP was slightly higher in iCJD than in sCJD, no significant differences in the distribution pattern of PrP between the two conditions were found (Figure 3C).

### Conformational stability immunoassay (CSI)

**Figure 4:**
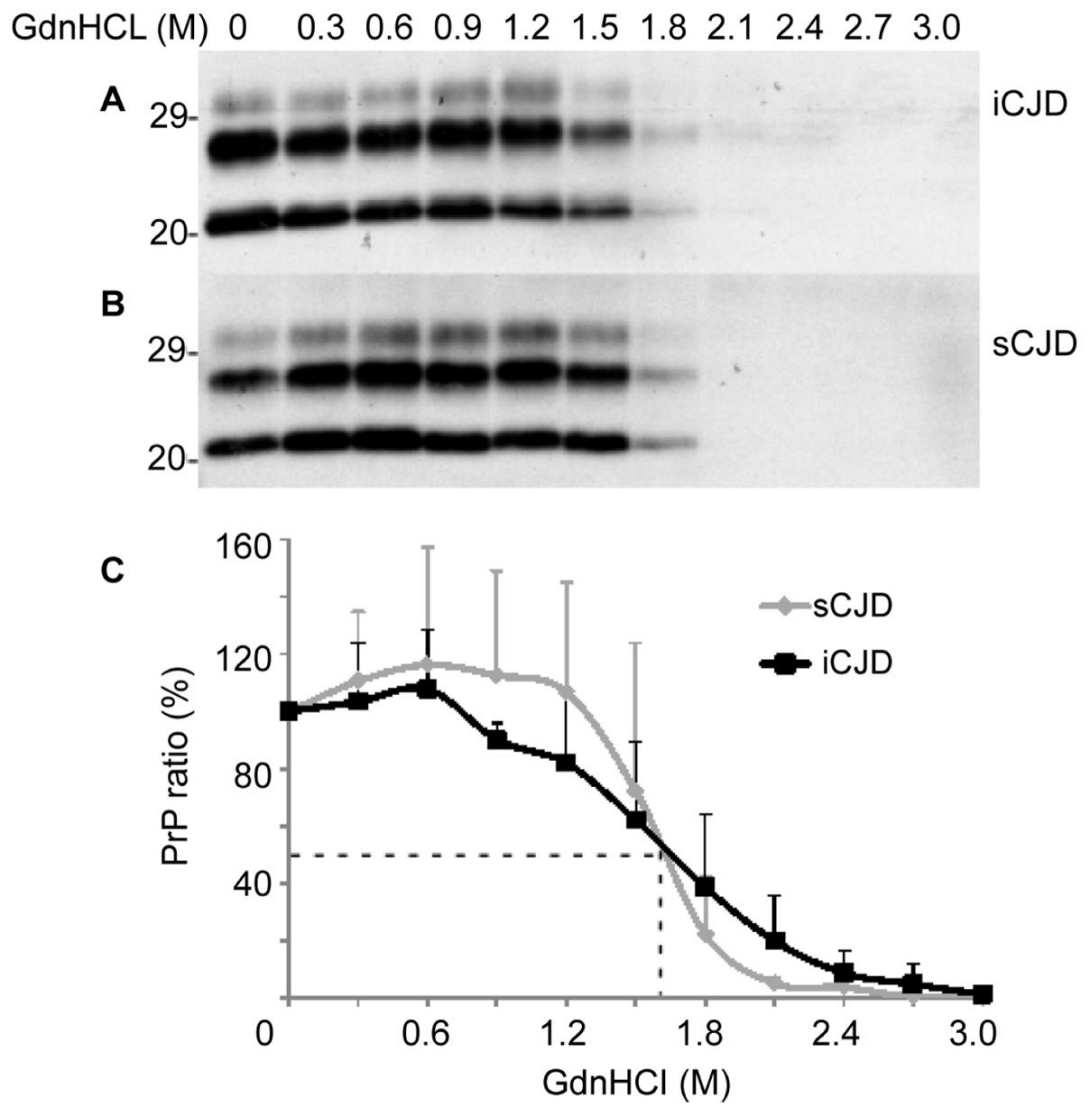
Conformational stability immunoassay of PrP^Sc^ **(A)** and **(B)** Western blots of PK-resistant PrP^Sc^ recovered from brain homogenates (A, iCJD; B, sCJD) following treatment with increasing concentrations of GdnHCl. **(C)** Levels of PK-resistant PrP^Sc^ from iCJD or sCJD as a function of increasing concentrations of GdnHCl. The dotted line represents PrP at 50% percentage of total PrP and corresponding concentration of GdnHCl (GdnHCl_1/2_). There was no significant difference in GdnHCl_1/2_ between iCJD (n=3) and sCJD (n=3).

CSI has been used to assess the stability of the PK-resistant PrP^Sc^ as a function of increasing concentrations of the denaturant GdnHCl that makes PrP^Sc^ PK-sensitive [19,20]. The GdnHCl concentration required to make half of the total PrP^Sc^ sensitive to PK digestion (GdnHCl_1/2_) is used as a measurement of the relative conformational stability of PrP^Sc^. We compared PrP^Sc^ from iCJD and sCJD using CSI to determine whether there is any difference in their conformational stability. We examined three cases for each CJD group. Their GdnHCl_1/2_ values were virtually identical (GdnHCl_1/2_= 1.65 ± 0.19 M) (Figure 4). The result suggests that the PrP^Sc^ species from both iCJD and sCJD have a similar conformational stability.

### Converting (or seeding) activity of PrP^Sc^ by protein misfolding cyclic amplification (PMCA)

**Figure 5:**
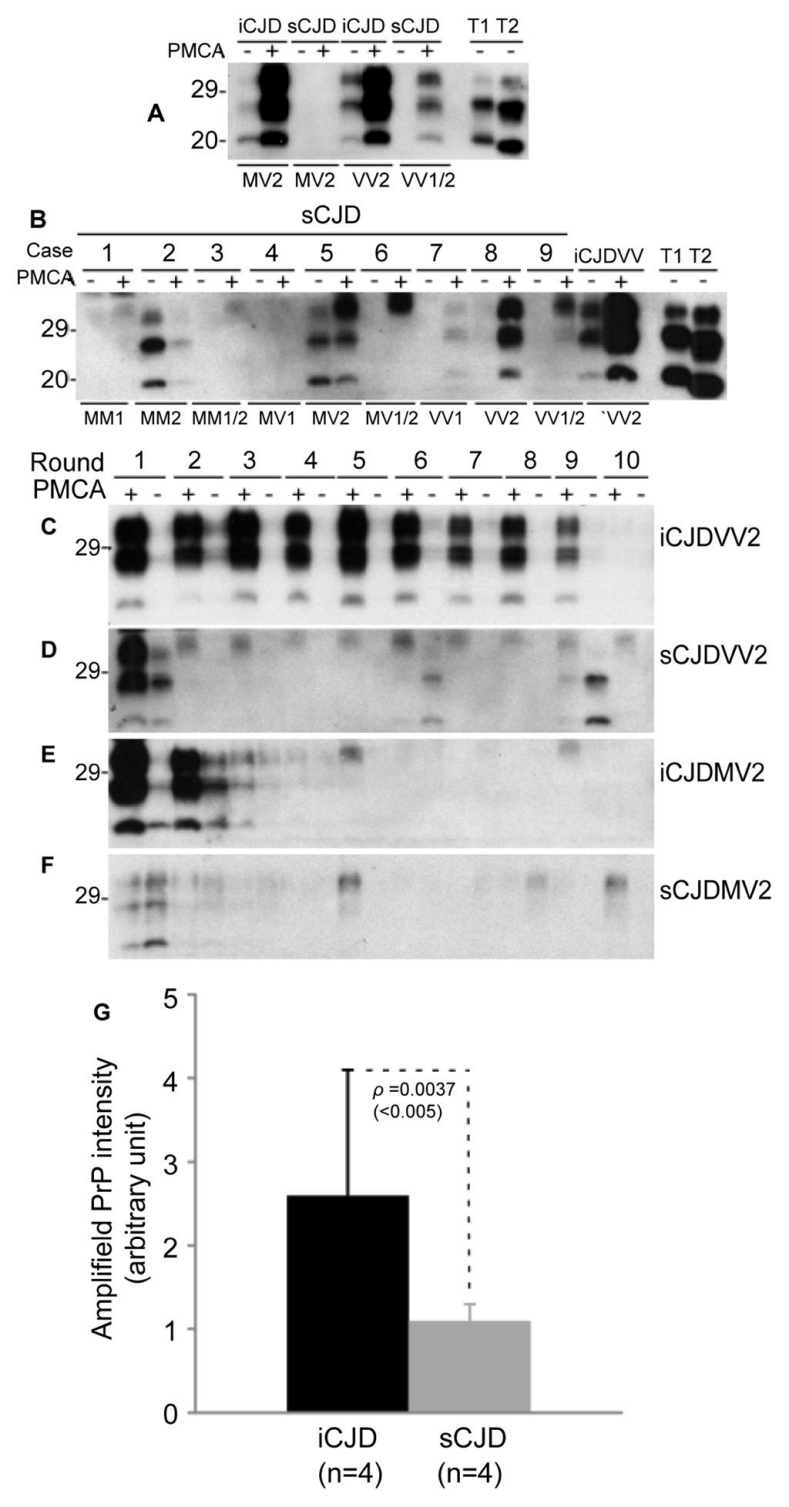
Converting activity of PrP^Sc^ by PMCA The amplification efficiency of PrP^Sc^ from iCJD or sCJD was compared based on the increased amount of PrP^Sc^ after PMCA in the presence of brain homogenates from Tg mice expressing human PrP-129M as a substrate. Amplified PK-resistant PrP^Sc^ was determined by Western blotting with 3F4. Samples without PMCA were used as controls. T1: PrP^Sc^ type 1; T2: PrP^Sc^ type 2. **(A)** Western blot analysis of amplified PK-resistant PrP^Sc^ from 2 iCJD (MV and VV) and 2 sCJD (MV2 and VV1-2) after PMCA for 18 h; **(B)** Western blot of amplified PK-resistant PrP^Sc^ from nine sCJD and one iCJDVV after PMCA for 18 h. From left to right 1: sCJDMM1; 2: sCJD MM2; 3: sCJDMM1-2; 4: sCJDMV1; 5: sCJDMV2; 6: sCJDMV1-2; 7: sCJDVV1; 8: sCJDVV2; and 9: sCJDVV1-2. **(C–F)** Western blot analysis of amplified PK-resistant PrP^Sc^ from ten rounds of serial PMCA with iCJDVV2 (C), sCJDVV2 (D), iCJDMV2 (E), and sCJDMV2 (F). **(G)** Quantitative analysis of amplification efficiency of PrP^Sc^ between iCJD (n=4) and sCJD (n=4). Amplified PrP^Sc^ was determined by using the PrP intensity detected in PMCA-treated samples to subtract the PrP intensity detected in untreated samples.

PMCA is a highly efficient cell-free PrP^Sc^ amplification assay. Using this approach, we compared the amplification efficiency of PrP^Sc^ from iCJD and sCJD. Given that most iCJD cases examined in our study were the MM genotype, we employed brain homogenates of humanized Tg mice expressing human PrP^C^-129M (Tg40h) as substrates. Compared to non-PMCA-treated samples, the intensity of PrP in PMCA-treated samples was significantly increased in two iCJD (MV2 and VV2) and one (sCJDVV2) of two sCJD cases (Figure 5A). No increase in the PrP intensity was found in the other sCJDMV2 case, suggesting that seeding activity of PrP^Sc^ in PrPC-129M substrates is greater in iCJD than in sCJD. To further investigate the effects of different genotypes at codon 129 and PrP^Sc^ types on the seeding activity, nine sCJD cases including 129MM, 129VV and 129MV genotypes and type 1 or 2 of PrP^Sc^ were examined. Amplification was detectable in the following sCJD cases: case 7 (VV1), case 8 (VV2), and case 9 (VV1-2), evidenced by an increase in the PrP intensity in PMCA-treated samples (Figure 5B). An increase in the intensity of only the upper band was observed in the sCJD cases 5 (MV2) and 6 (MV1). No increase in seeding activity was detected for case 1 (MM1), case 2 (MM2), case 3 (MM1-2) and case 4 (MV1). So, seeding activity seems to be determined largely by the genotype and PrP^Sc^ type in the PMCA using brain homogenates of humanized Tg40h mice expressing PrP^C^-129M as substrates.

It has been demonstrated that hamster PrP^Sc^ can be amplified by infinite rounds using serial PMCA (sPMCA) [15], indicating that not only sPMCA itself is highly efficient but also the seeding activity of hamster prion strain is extremely high. Using this method, we tested the limit of possible rounds allowing the amplification of PrP^Sc^ from iCJD and sCJD. PK-resistant PrP was detectable until round 9 with the PrP^Sc^ seeds from iCJDVV2 while it was detectable only in the first round with the PrP^Sc^ seeds from sCJDVV2 (Figures 5C and 5D). PrP^Sc^ from iCJDMV2 was amplified until round 3 while PrP^Sc^ from sCJDMV2 exhibited no amplification even in the first round (Figures 5E and 5F). Again, the presence of two V-alleles exhibited a higher amplification efficiency compared to cases carrying a single V-allele. We also calculated the seeding efficiency of PrP^Sc^ by measuring the amount of amplified PrP^Sc^, using the intensity of PrP in PMCA-treated samples to subtract intensity of untreated PrP. The intensity of amplified PrP was significantly greater in iCJD (n = 4) than in sCJD (n=4) (p=0.0037 <0.005) (Figure 5C).

### Mapping of PK-resistant C-terminal fragments of PrP (PrP-CTF12/13)

**Figure 6:**
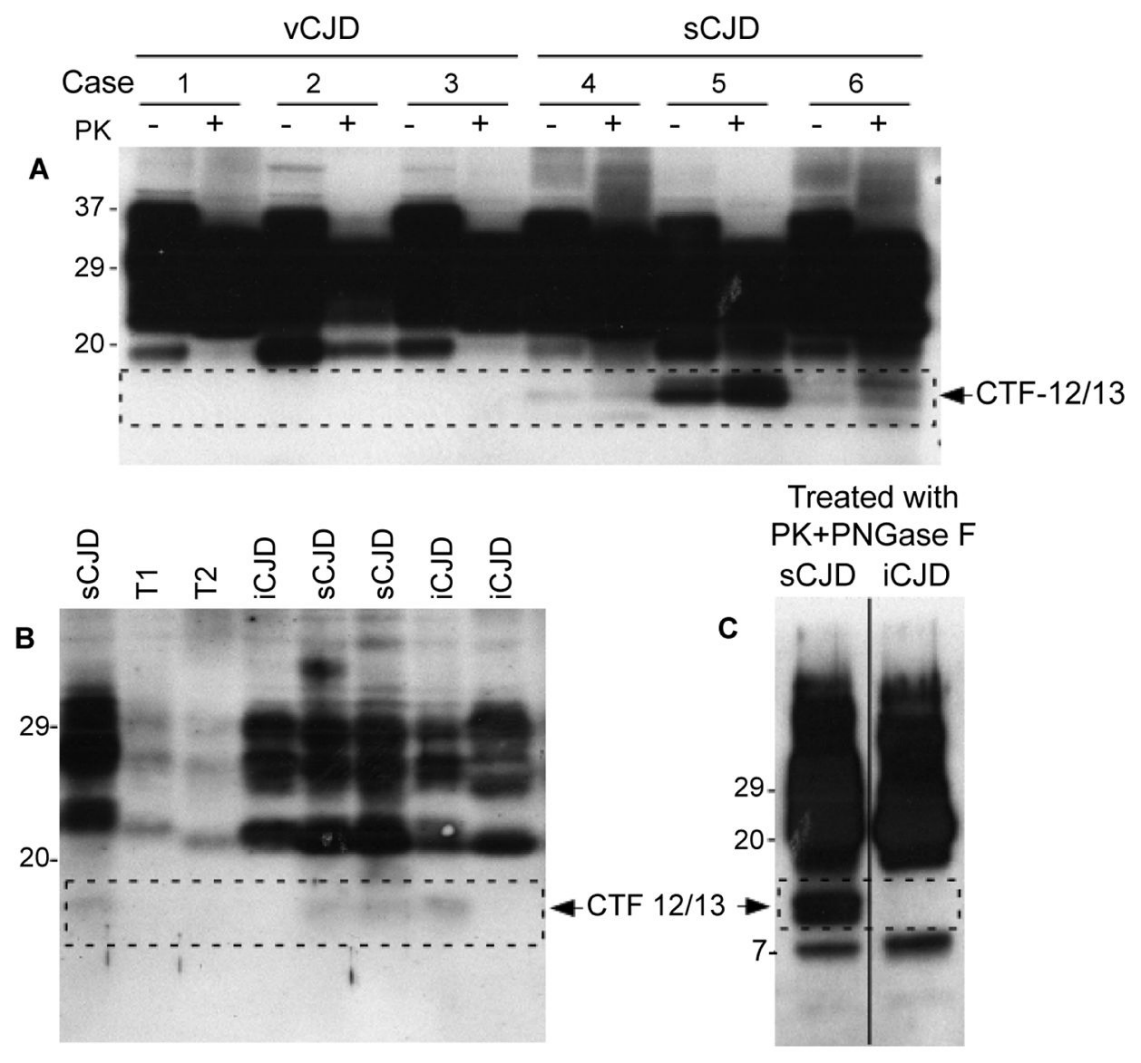
PK-resistant PrP C-terminal fragments (PrP-CTF12/13) **(A)** Detection of PrPCTF12/13 in vCJD and sCJD by Western blotting with anti-C antibody against human PrP220-231. While the PrP-CTF12/13 fragments were detectable in all three sCJD cases, they were undetectable in all three vCJD (see dotted line rectangle). **(B)** Western blot analysis of PrPCTF12/13 in iCJD and sCJD. All three sCJD cases exhibited PrP-CTF12/13. In contrast, only one out of three iCJD had PrP-CTF12/13 (dotted line rectangle). **(C)** Western blot analysis of PrP-CTF12/13 in PrP^Sc^ from sCJD or iCJD, after treatment with PK and PNGase F (dotted line rectangle), confirmed the absence of PrP-CTF12/13 fragments.

We previously identified two PK-resistant C-terminal fragments of PrP called PrP-CTF12/13 in sCJD, a spontaneous form of prion disease [23]. Whether acquired form of prion disease including vCJD and iCJD also contains PrP-CTF12/13 or not remains to be further confirmed, although similar PrP C-terminal fragments were reported undetectable [24,25]. We first compared three vCJD and three sCJD cases after PK-treatment by Western blotting probing with anti-C antibody termed 2301 that is directed against a PrP C-terminal domain between residues 220 and 231 [23]. As expected, PrP-CTF12/13 fragments migrating at about 12 kDa and 13 kDa were detectable in all three PK-treated sCJD cases (Figure 6A). In contrast, no PrP-CTF12/13 fragments were detected in all three vCJD samples (Figure 6A). Next, PrP-CTF fragments in iCJD cases were examined. Although all three sCJD cases revealed PrP-CTF, two out of three iCJD showed no PrP-CTF fragments (Figure 6B). To confirm it, we treated brain homogenates from sCJD and iCJD with PK plus PNGase F prior to detection of PrP-CTF, a treatment that can significantly increase the fragments. Although much intense PrP-CTF12/13 fragments were detected in the PK/PNGase F-treated sCJD sample, there was no PrP-CTF12/13 detected in the treated iCJD sample except for a PrP-CTF7-8 that was detected in both (Figure 6C). In total, 6 out of 8 iCJD cases examined were observed to have no PrP-CTF fragments while all 9 sCJD cases examined exhibited the two small PK-resistant C-terminal fragments. So, PrP-CTF12/13 fragments were undetectable in most of iCJD (and all vCJD) cases, which is different from sCJD.

### Infectivity of PrP^Sc^

Since PrP^Sc^ with 129MM from both iCJD and sCJD exhibited no seeding activity by PMCA with humanized Tg40h, we further compared the infectivity of PrP^Sc^ from iCJD and sCJD by inoculating them into the same line of Tg40h mice (n=32). The mice were inoculated with brain homogenates from two iCJD cases (cases 1 and 2 shown in Figure 2A) and two sCJD cases (cases 9 and 10 shown in Figure 2A) cases, respectively (Table 1). The mice inoculated with brain homogenates from the two separate iCJD cases had incubation time of 181 ± 3 days or 170 ± 7 days, respectively. The mice inoculated with brain homogenates from two sCJD cases had incubation time of 185 ± 5 days or 185 ± 3 days, respectively (Table 1). Regardless of type of the original iCJD or sCJD inocula employed, all diseased Tg mice exhibited similar clinical signs, including hunched back, ruffled fur, rigid tails, and slow movement. The attack rate was 100% in both groups of mice. No differences in incubation time and transmission rate were observed between iCJD and sCJD (Table 1).

**Figure 7:**
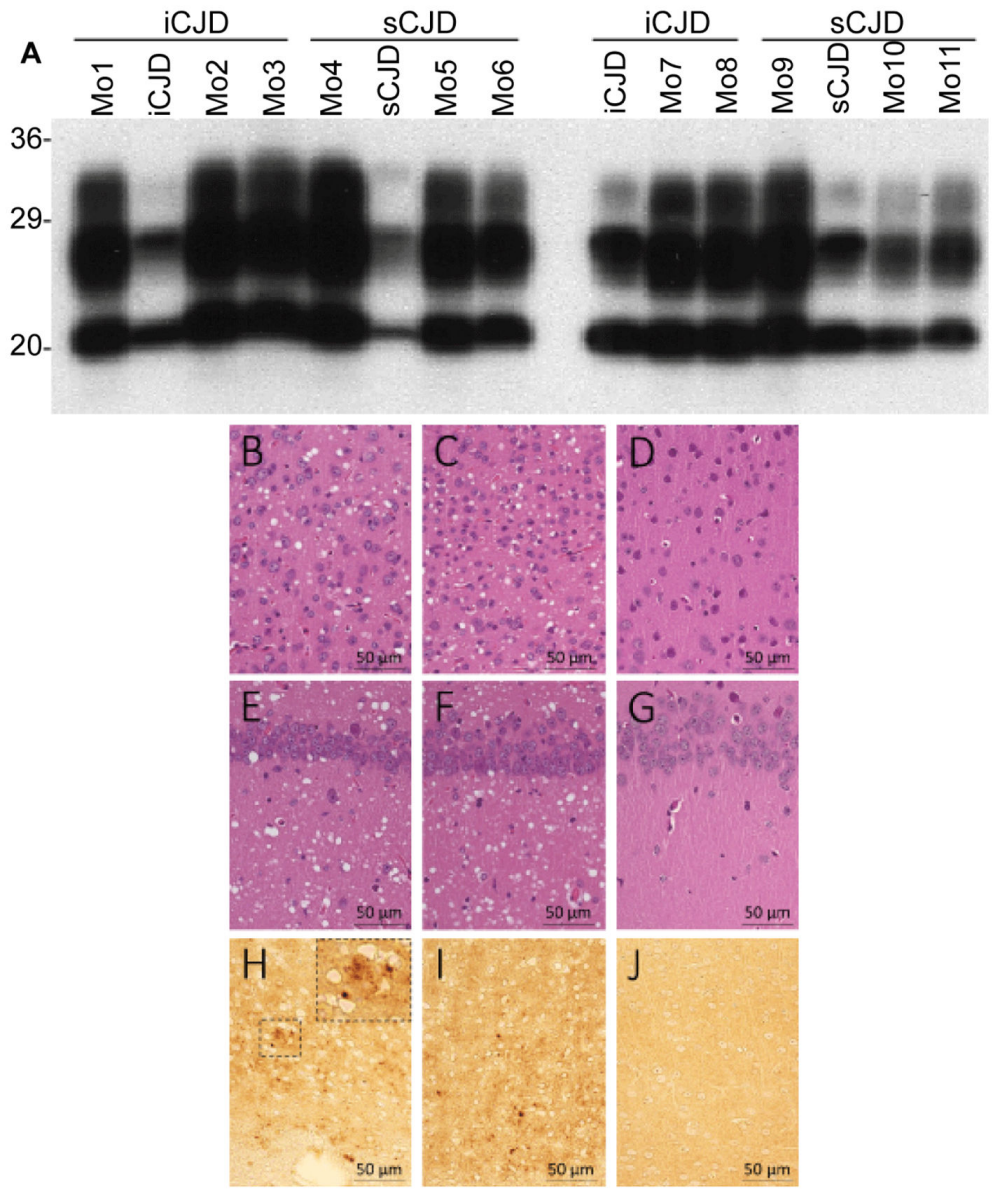
Western blot, histopathology and immunohistochemistry of Tg40h mice inoculated with iCJD or sCJD prions **(A)** Western blot of PK-resistant PrP^Sc^ from brain homogenates (BH) of Tg40h mice and original inocula. As shown in Table 1, groups of eight Tg40h mice expressing human PrP^C^-129M were inoculated with BH from two iCJD or two sCJD harboring PrP^Sc^ type 1, respectively. The mice reproduced the same PrP^Sc^ type 1 and the same glycoform ratio as that of the original inocula. **(B–G)** Hematoxylin–eosin staining exhibits spongiform degeneration in the cerebral cortex **(B-C)** and hippocampus **(E–F)** in the mice inoculated with iCJD **(B–E)** or sCJDMM1 **(C–F)**. No SD is observed in the mice inoculated with 1X PBS (D: cerebral cortex; G: hippocampus). H-J: Immunohistochemistry shows a granular PrP immunostaining and microplaque-like formations in the cerebral cortex of the mice inoculated with iCJD **(H)**, sCJDMM1 **(I)** but not in mice inoculated with 1X PBS; antibody: 3F4. Overall, no differences in neuropathological changes were seen between Tg40h mice inoculated with iCJD or sCJD brain preparations.

We examined the PrP^Sc^ gel profile in Tg mice inoculated with either iCJD or sCJD PrP^Sc^. No difference was detected in the PK-treated PrP^Sc^ gel profile within the same or the two different groups of inoculated Tg mice (Figure 7A). Moreover, the PK-resistant PrP^Sc^ from the inoculated Tg40h mice showed the same electrophoretic profile as those of the original iCJD and sCJD inocula. Formalin-fixed brain sections of Tg mice were also examined with hematoxylin & eosin (H&E) staining and immunohistochemistry (IHC) with 3F4. At histopathological examination, Tg40h mice inoculated with brain homogenates from iCJD or sCJD showed a similar distribution of the spongiform degeneration (Figures 7B through 7G), which was more severe in the cerebral cortex, hippocampus and basal ganglia compared to the thalamus and brain stem; the cerebellum was intact. Immunohistochemistry for PrP staining was also similar in both groups of mice and revealed a very fine PrP staining with small granular and microplaque-like formations in the cerebral cortex, hippocampus and basal ganglia and a more intense staining, occasionally with plaque-like formations, in the thalamus (Figures 7H through 7J). There was also a minimal PrP staining of both upper and lower brain stem nuclei and virtually no PrP staining in the cerebellum.

## Discussion

In the current study, we compared similarities and differences in the physicochemical and biological features of PrP^Sc^ in iCJD and sCJD using a combination of various sophisticated approaches. No significant differences were observed in gel mobility, glycoform ratios, oligomeric state, conformational stability, and infectivity of PrP^Sc^ between the two conditions. However, two important features were identified in most of the iCJD cases – the absence of PrP-CTF12/13 and the presence of high seeding activity – that could distinguish iCJD from sCJD in ambiguous clinical situations.

PrP-CTF12/13 fragments were first identified in sporadic CJD [23]. Like PrP27–30, they derive from both glycosylated and unglycosylated forms. Small amounts of the fragments are detectable prior to PK-digestion *in vitro*, suggesting that they are present in the brain intrinsically. The amounts of the fragments are significantly increased upon treatment with PK and PNGase F *in vitro*. The unglycosylated PrP-CTF12/13 migrate at 12 and 13 kDa and their N-termini start at residues 162/167 and 154/156, respectively [23]. PrP-CTF12/13 are about half of the size of PrP27–30. It has been proposed that PrP-CTF12/13 fragments are likely to originate from a subpopulation of PrP^Sc^ distinct from the PrP^Sc^ species which generates PrP27–30 [23]. The origin and significance of PrP-CTF12/13 in the pathogenesis of prion disease remain to be determined. In the present study, we found that 75% (6 out of 8) iCJD cases examined had no PrP-CTF12/13 while the small fragments were detected in all nine sCJD and the two remaining iCJD cases. Moreover, PrP-CTF12/13 fragments were also not detectable in all three vCJD cases examined. Notably, our study on iCJD and vCJD are consistent with a previous observation by Satoh et al. and Notari et al. [24,25]. In eight dura mater graft associated CJD (dCJD) cases, Satoh et al. reported a C-terminal PrP fragment of 11–12 kDa termed fPrP11-12, a fragment that could be equivalent to or be the same as the PrP-CTF12 identified by our studies [ref 23 and the current study] and that was detected only in three cases without PrP plaques (np-dCJD) but not in other five cases with PrP plaques (p-dCJD) [24]. Moreover, this C-terminal fragment was also detected in fCJD linked to E200K or M232R PrP mutation, whereas it was not detectable in vCJD [24]. In contrast with an intermediate fragment PrP7-8 that is always associated with PrP plaques in familial prion disease GSS [26], interestingly, the C-terminal fragments seem to be associated with prion diseases that do not form PrP plaques. PrP-CTF12/13 fragments have been observed in all sCJD examined by us in spite of variable levels [ref 23 and the present study]. However, fPrP11-12 was undetectable in two sCJDVM2 and one sCJDVV2 examined by Satoh et al. [24].

Given that both vCJD and iCJD are acquired while sCJD and familial CJD (fCJD) are spontaneous, the lack of PrP-CTF12/13 in the formers suggests that acquired and spontaneous prion diseases may have distinct prion formation pathways. The lack of PrP-CTF12/13 or fPrP11-12 in vCJD and most iCJD including p-dCJD may be associated with a prion formation pathway that is different from a pathway present in other types of prion diseases that have no plaque deposition but accompany with the small fragments. There seem to be two types of pathways of prion formation and process. The first one is associated with acquired prion diseases in which PrP^Sc^ is formed on the cell surface and released directly into extracellular space to form aggregates without any intracellular process. The second is associated with spontaneous prion diseases in which PrP^Sc^ is formed in the intracellular endosome-lysosome system or after formed on the cell surface, PrP^Sc^ is transported into the intracellular endosome-lysosome system. It is conceivable that the most imported exogenous PrP^Sc^ in iCJD is propagated only on the cell surface and then formed aggregates are directly released into extracellular space. The PrP aggregates may not be transported into the endosome-lysosome pathway. On the other hand, in spontaneous prion diseases such as sporadic or hereditary CJD, PrP^Sc^ may be formed either in the endosome-lysosome system or on the cell surface but it is transported into the endosome-lysosome system. The endosome-lysosome system may be the place where PrP-CTF12/13 fragments are formed. Indeed, it has been proposed that misfolded PrP^Sc^ aggregates formed on the cell surface are either released into extracellular space or packaged in vesicles and then transported into the endosome-lysosome pathway [27]. However, it is unclear why iCJD also exhibits both types of cases either with or without the PKresistant C-terminal fragments [ref 24 and this study]. In addition to dCJD [24], congophilic amyloid plaques were also detected in both gray and white matters of the cerebrum and cerebellum in an iCJD case associated with cadaver-derived human growth hormone [28]. It may explain why there were also two types of cases with or without PrP-CTF12/13 in our study in which there were 7 out of 8 cases associated with growth hormone. Moreover, whether vCJD exclusively exhibits no PrP-CTF12/13 remains to be further determined with more cases since only three vCJD cases were examined in our study.

The second feature of PrP^Sc^ from iCJD found in the current study is its high seeding activity compared to sCJD by the PMCA assay. Amplified PrP^Sc^ from sCJDVV2 examined was only detectable in the first round of PMCA. No amplification was observed in the second or more rounds. In contrast, PrP^Sc^ from iCJD (MV2 or VV2) was amplifiable mostly by the first two rounds of PMCA, although no amplification was detected after two rounds except for PrP^Sc^ from iCJDVV2 that was amplifiable even at round nine. It has been well-demonstrated that hamster PrP^Sc^ 263K can be amplified infinitely [15]. So, in general, seeding activity of human prions is lower compared to that of hamster prions. In addition, the amplification efficiency of PrP^Sc^ detected in the first round was significantly greater in iCJD than in sCJD. All these findings suggest that the seeding activity and amplification efficiency of PrP^Sc^ are increased in iCJD than in sCJD. It supports a possibility that the intra-species prion transmission from human-to-human occurring in iCJD also causes prion adaptation to increase propagation efficiency probably by conformational change. This possibility is also supported by a recent transmission study by Bishop et al. [29].

However, it is worth noting that amplification and increased seeding activity of PrP^Sc^ by PMCA were highly dependent on genotype and protein type of PrP^Sc^ from iCJD using the PrP^C^- 129M substrate. First, the PrP^Sc^ V-allele seems to be highly amplifiable than the M-allele even in the PrP^C^-129M substrate. For instance, amplification was mainly observed with iCJDVV2 and sCJDVV2. Amplification was only observed with iCJDMV2 but not with sCJDMV2. Regardless of iCJD or sCJD, no amplification was detected for type 1 or 2 PrP^Sc^-129MM. This is surprising since the best transmission and amplification efficiency of CJD PrP^Sc^ was observed when the 129-MV polymorphism matches between PrP^Sc^ seed and PrP^C^ substrate *in vivo* and *in vitro* [29,30]. Possibilities cannot be excluded that this discrepancy between ours and Jones et al. [30] resulted from the different substrates used in the two studies that were from different lines of Tg mice and other different experimental conditions. However, although no amplification was observed by PMCA, iCJDMM1 and sCJDMM1 exhibited similar infectivity after inoculated into the same line of Tg40 mice. The mechanisms underlying the unorthodox polymorphism-dependent seeding activity and amplification need to be investigated in the future. Moreover, it will be interesting to further determine whether the high seeding activity of V-allele found by our PMCA assay is consistent with the high prevalence of PrP^Sc^-129VV (65% VV vs. 23% MV vs. 12% MM) in variably protease-sensitive prionopathy, an atypical human prion disease identified by our previous study [31]. Second, the seeding activity was higher with sCJDVV2 than with sCJDVV1 or sCJDVV1-2, suggesting that the type 2 PrP^Sc^ is readily amplified *in vitro* compared to type 1. Finally, all iCJDVV2, iCJDMV2, or sCJDVV2 were amplified to a type 1-like PK-resistant PrP^Sc^ by PMCA in the PrP^C^-129M substrate in this study. Our recent work has demonstrated that PrP^Sc^ from the same iCJDVV2 can be amplified faithfully in the PrP^C^-129V substrate from our newly-generated Tg (HuPrP-129V) mice expressing human PrP^C^-129V [21]. The combination of these results indicated that PrP^Sc^ type 1 may be preferentially amplified in PrP^C^-129M substrate while PrP^Sc^ type 2 may be preferentially amplified in PrP^C^-129V substrate. Our findings with PMCA are in agreement with the prevalence of sCJD in which PrP^Sc^ type 1 is mainly associated with the MM genotype, whereas PrP^Sc^ type 2 is mainly associated with the VV genotype [32].

## Figures and Tables

**Table 1:** Prion transmission in transgenic mice.

Prion inoculum	Mice	Incubation time (day, SD)	Transmission rate
iCJD case 1	Tg 40h	181 ± 3	8/8
iCJD case 2	Tg 40h	170 ± 7	8/8
sCJD case 1	Tg 40h	185 ± 5	8/8
SCJD case 2	Tg 40h	185 ± 3	8/8
